# Genome sequencing broadens the range of contributing variants with clinical implications in schizophrenia

**DOI:** 10.1038/s41398-021-01211-2

**Published:** 2021-02-01

**Authors:** Bahareh A. Mojarad, Yue Yin, Roozbeh Manshaei, Ian Backstrom, Gregory Costain, Tracy Heung, Daniele Merico, Christian R. Marshall, Anne S. Bassett, Ryan K. C. Yuen

**Affiliations:** 1 Genetics and Genome Biology, The Hospital for Sick Children, Toronto, ON Canada; 2 Ted Rogers Centre for Heart Research, Cardiac Genome Clinic, The Hospital for Sick Children, Toronto, ON Canada; 3 Division of Clinical and Metabolic Genetics, The Hospital for Sick Children, Toronto, ON Canada; 4 Clinical Genetics Research Program, Centre for Addiction and Mental Health, Toronto, ON Canada; 5 The Dalglish Family 22q Clinic for Adults with 22q11.2 Deletion Syndrome, Toronto General Hospital, University Health Network, Toronto, ON Canada; 6 Deep Genomics Inc., Toronto, Ontario and The Centre for Applied Genomics (TCAG), The Hospital for Sick Children, Toronto, ON Canada; 7 Paediatric Laboratory Medicine, Genome Diagnostics, The Hospital for Sick Children, Department of Laboratory Medicine and Pathobiology, University of Toronto, Toronto, ON Canada; 8 Department of Psychiatry, University of Toronto, Toronto General Hospital Research Institute and Campbell Family Mental Health Research Institute, Toronto, ON Canada; 9 Department of Molecular Genetics, University of Toronto, Toronto, ON Canada

**Keywords:** Clinical genetics, Psychology

## Abstract

The range of genetic variation with potential clinical implications in schizophrenia, beyond rare copy number variants (CNVs), remains uncertain. We therefore analyzed genome sequencing data for 259 unrelated adults with schizophrenia from a well-characterized community-based cohort previously examined with chromosomal microarray for CNVs (none with 22q11.2 deletions). We analyzed these genomes for rare high-impact variants considered causal for neurodevelopmental disorders, including single-nucleotide variants (SNVs) and small insertions/deletions (indels), for potential clinical relevance based on findings for neurodevelopmental disorders. Also, we investigated a novel variant type, tandem repeat expansions (TREs), in 45 loci known to be associated with monogenic neurological diseases. We found several of these variants in this schizophrenia population suggesting that these variants have a wider clinical spectrum than previously thought. In addition to known pathogenic CNVs, we identified 11 (4.3%) individuals with clinically relevant SNVs/indels in genes converging on schizophrenia-relevant pathways. Clinical yield was significantly enriched in females and in those with broadly defined learning/intellectual disabilities. Genome analyses also identified variants with potential clinical implications, including TREs (one in *DMPK*; two in *ATXN8OS*) and ultra-rare loss-of-function SNVs in *ZMYM2* (a novel candidate gene for schizophrenia). Of the 233 individuals with no pathogenic CNVs, we identified rare high-impact variants (i.e., clinically relevant or with potential clinical implications) for 14 individuals (6.0%); some had multiple rare high-impact variants. Mean schizophrenia polygenic risk score was similar between individuals with and without clinically relevant rare genetic variation; common variants were not sufficient for clinical application. These findings broaden the individual and global picture of clinically relevant genetic risk in schizophrenia, and suggest the potential translational value of genome sequencing as a single genetic technology for schizophrenia.

## Introduction

Schizophrenia is a serious and disabling neuropsychiatric disorder that affects about 1% of the general population. Despite inherent heterogeneity, a century of research has provided strong evidence of genetic predisposition, and statistical modelling has consistently indicated high heritability^1,2^. However, discerning specific genetic risk factors for individuals with schizophrenia awaited technological advances in molecular genetics. Studies using first genome-wide chromosomal microarray (CMA) and then whole-exome sequencing (WES) have provided initial clues to the underlying genetic architecture of schizophrenia. These include contributions of rare (population frequency ≤0.1%) copy number variants (CNVs), other rare damaging and deleterious variants, common (population frequency >1%) single-nucleotide polymorphisms (SNPs), and evidence for long-suspected polygenicity^3–6^. Although the rare high-impact variants identified are often shared with other neurodevelopmental disorders (NDDs)^7,8^, in contrast to autism spectrum disorder (ASD), intellectual disability (ID) and epilepsy, relatively few genetic findings for schizophrenia have reached the clinic^1,9,10^.

Whole-genome sequencing (WGS) captures most forms of genetic variation across the genome in a single assay, surpassing the capabilities of CMA and WES combined^11^. Furthermore, recent technical advances in WGS techniques and analyses allow for the genotyping of more complex genetic variation, such as tandem repetitive DNA elements, throughout the genome, not readily detectable using other sequencing techniques^12^. The pathogenicity of large expansions of tandem DNA (in particular trinucleotide) repeats, has been extensively studied in over 40 genetic disorders, most of which are neurological but sometimes include psychosis^13^. Clinical observations of increased severity and/or younger age at the onset across successive generations historically suggested anticipation in schizophrenia, supporting the possible involvement of repeat expansions^14,15^. However, the technologies and methodologies available to detect such repetitive DNA elements before now were limited.

In the current study, we applied WGS to a well-characterized community-based cohort of unrelated adults with schizophrenia. Our aim was, for the first time using a clinical lens and WGS data, to simultaneously detect multiple classes of genome-wide rare, high-impact genetic variants (including CNVs, single-nucleotide variants (SNVs), small insertions and deletions (indels), structural variants (SVs), and tandem repeat expansions (TREs), and assess for schizophrenia-related polygenic risk, while investigating possible phenotype correlations. Here, we defined high-impact variants as those with clinical relevance to schizophrenia or with potential clinical implications. Using this approach, we underscore the importance of thorough genome analyses in the identification of variants with potential clinical implications in individuals with schizophrenia, with or without molecular findings from routine CMA. This study thus expands on previous WGS studies of schizophrenia (Supplementary Table S1) to serve as an initial step in demonstrating the potential value of WGS as a single clinically relevant genetic technology for schizophrenia.

## Methods

### Study design

The 259 participants comprise a subset of a larger well-characterized cohort of unrelated adults who: (i) met standard diagnostic (DSM-5) criteria for schizophrenia or schizoaffective disorder, (ii) were of European descent, and (iii) were previously examined for the presence of rare CNVs (≥10 kb in size) using CMA^16,17^. Participants were ascertained from Canadian community mental health clinics and included individuals with schizophrenia across the IQ spectrum; details of the ascertainment strategy are described elsewhere^16,17^. A priori, individuals with 22q11.2 microdeletions were excluded, as this established genetic subtype of schizophrenia is studied with WGS through a separate research initiative^18^. Also by design^19^, 136 (52.5%) of the individuals included in this study had broadly defined schizophrenia-relevant rare CNVs^17^ (Supplementary Tables S2, S3), 26 (10%) of whom had 28 CNVs previously classified as clinically relevant (pathogenic/likely pathogenic, Supplementary Table S2)^16,17^. By including individuals with rare CNVs (10% with pathogenic CNVs), we undertook a conservative approach, interrogating for other potentially clinically relevant variants beyond well-studied CNVs.

### Ethics statement

This study was approved by the Research Ethics Board at the Centre for Addiction and Mental Health (CAMH) (151/2002-02) and other local REBs^16,17^. Written informed consent was obtained for all participants^16,17^.

### Assessment of the pathogenicity of rare variants (SNVs, indels, SVs, CNVs)

All rare (defined as population-based maximum allele frequency ≤0.01) exonic and exonic-splicing SNVs and indels, SVs, and CNVs were analyzed for their potential pathogenicity. Population allele frequency of each variant was derived from genomes included in ExAC, 1000 Genomes Project, gnomAD and gnomAD SV databases^20–23^. Probability of loss-of-function (LoF) intolerance was measured by the upper bound of a Poisson-derived confidence interval around the ratio of the observed/expected number of LoF variants in every gene, derived from gnomAD (v2.1.1) and represented by LoF observed/expected upper bound fraction (LOEUF) score^20^. LoF variants were defined as stop-gains, frameshift indels, and splice-site variants. Rare nonsynonymous variants with high predicted scores in 5 of 8 commonly used in silico algorithms [CADD (≥15), SIFT (≤0.05), PolyPhen2 HVAR (≥0.90), Provean (<-2.5), ma (≥1.90) and mt (≥0.5) scores, PhyloPMam (≥2.30) and PhyloPVert (≥4.0)] were considered as deleterious and were further assessed for pathogenicity^24^. Given the evidence for genetic overlap between schizophrenia and other major NDDs, we conservatively considered only loci and genes as potentially associated with schizophrenia if they had been implicated in any NDD (e.g., ID or ASD), and their implicated pathways (Supplementary information)^7,8,25–38^. Pathogenicity of rare SVs was assessed using their predicted damaging or deleterious effects on genes implicated in NDDs. In this study, for CNVs, SNVs and indels, we considered only pathogenic and likely pathogenic variants as potentially clinically relevant^24^ and contributing to the expression of schizophrenia, as adjudicated for NDDs.

### Detection and independent confirmation of disease-associated tandem repeats using genome sequence data

To assess the presence of high-impact TREs in the genomes of our schizophrenia cohort, we collected data for 45 tandem repeat loci with known clinical associations, predominantly with neurological disorders (Supplementary Table S4). We used ExpansionHunter v3.0.2 to genotype these genomic repeat loci^39^, and selected TREs larger than the described pathogenicity threshold for each locus for further characterization (Supplementary information). (https://github.com/Illumina/ExpansionHunter)^40^. Rare TREs were classified as variants with a high-impact if the predicted size for the larger allele in each individual exceeded the disease-causing threshold for their loci^13^.

### Clinical/demographic variables

We considered the following clinical/demographic variables for analyses: sex, presence or absence of family history of schizophrenia/psychotic illness, ID (broadly defined as borderline to moderate ID and non-verbal learning disability)^16^, syndromic features, and age at onset of schizophrenia (categorized as <18 years (“early”) or ≥18 years); details in Supplementary information, Supplementary Table S5, and as previously described^16,17^. To assess these variables with respect to clinically relevant rare variant burden, we used a stringent definition, including only pathogenic CNVs and SNVs/indels, defined as above, but not TREs, following well-established guidelines^24^.

### Additional exploratory analyses

In addition to the primary focus on clinically relevant rare variants, we explored the possible role in our cohort of research-based genetic findings for schizophrenia, e.g., from exome sequencing and SNP-based studies.

### Assessment of variants in putative schizophrenia-risk genes

To assess research-based SNV findings, we examined our cohort for all types of rare SNVs in ten genes reported to meet genome-wide significance for schizophrenia from recent meta-analysis results of exome sequencing data from the Schizophrenia Exome Sequencing Meta-analysis (SCHEMA) consortium (https://schema.broadinstitute.org/)^40^.

### Schizophrenia polygenic risk quantification

To assess the role of aggregate common variant background (polygenic risk score, PRS) for schizophrenia, we used the training dataset provided by the 2014 PGC schizophrenia meta-analysis (Schizophrenia Working Group of the Psychiatric Genomics Consortium) to generate individual risk profile scores for our cohort^3^. There was no overlap of individuals in the training dataset with our schizophrenia cohort (Supplementary Fig. S1).

### Non-psychiatric controls

We used comparable WGS data available from a previously published study of tetralogy of Fallot (TOF) and related congenital cardiac disease^19^ as a non-psychiatric control group to evaluate individual gene rare SNV findings and PRS results. After excluding seven genomes from individuals with TOF and a history of major neuropsychiatric conditions (e.g., ASD, psychotic mood disorder), data were available from 225 of the 232 individuals in this TOF cohort^19^ (Supplementary information).

## Results

Demographic and clinical features of the community-based cohort of 259 unrelated individuals with schizophrenia studied with genome sequencing are presented in Supplementary Table S5. The genomes sequenced had an average of 98.1% of bases covered by at least >1×, and an average mean depth of coverage of 38.42× (Supplementary Tables S6, S7). Restricting to exonic rare (allele frequency ≤1%) variants, we detected on average 271.7 SNVs, 20.1 indels, 2.89 SVs, and 3.33 CNVs (≥10Kb) per genome, consistent with expectations from previous WGS analyses of other samples^41^.

### WGS enables simultaneous identification of multiple rare exonic variants of potential clinical relevance to schizophrenia

**Fig. 1:**
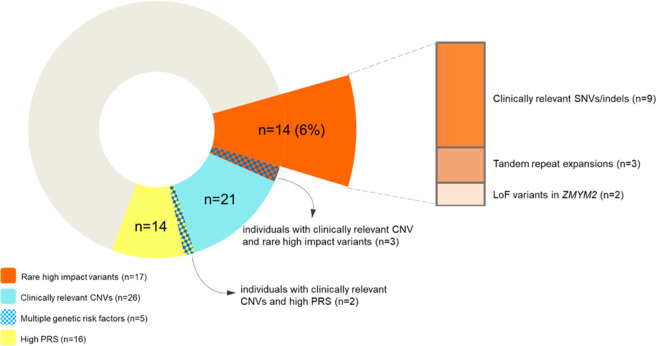
Schematic representation of the identified contributions of rare high-impact variants with potential clinical implications to schizophrenia. The overall “doughnut” graph indicates the study design that included 26 individuals (Supplementary Table S2) with pathogenic/likely pathogenic rare copy number variants (CNVs; blue sections, including five with other reported genetic risk factors indicated by blue checkered overlay). Red sections indicate the total 17 individuals identified to have other types of rare high-impact variants proposed to have potential clinical relevance for schizophrenia; 14 of these, representing 6% of individuals without pathogenic CNVs, are also shown with detailed breakdown of variant types in a bar graph on the right. This shows nine individuals with rare SNVs/indels, and three with CTG tandem repeat expansions (TREs), deemed to have potential clinical implications; also shown are two individuals with ultra-rare LoF variants in *ZMYM2*, proposed here as a putative schizophrenia-candidate gene. One other individual with an ultra-rare LoF variant in *ZMYM2*, and two individuals with clinically relevant rare SNVs/indels (Tables 1, 3), also had a pathogenic CNV (blue checkered overlay on red section of doughnut graph). Also shown (yellow sections) are 16 individuals belonging to the top twentieth percentile of schizophrenia-PRS (Supplementary Fig. S7); note that schizophrenia-PRS has not yet reached proposed clinical relevance.

WGS identified several types of rare exonic variants of potential clinical relevance in this schizophrenia cohort. Importantly, the WGS pipeline identified 100% of the 28 rare CNVs in 26 individuals that were previously reported as clinically relevant (pathogenic/likely pathogenic)^16,17^ (Fig. 1, Supplementary Tables S2, S3). No rare SVs<10Kb met criteria as pathogenic/likely pathogenic.

In eleven individuals (4.3%), WGS also identified clinically relevant SNVs and indels with predicted deleterious effects on loss-of-function (LoF)-intolerant genes previously associated with schizophrenia-related NDDs (Methods and Table 1). There were five frameshift indels, three nonsense (stopgain), and three deleterious missense variants identified in ten genes: eight autosomal and two X-chromosome (both in females) (Table 1). Notably, two of the 11 individuals, both with missense variants, also had a clinically relevant 16p11.2 microduplication associated with increased schizophrenia risk (Table 1, Supplementary Tables S2 and S3)^17^. We thus propose clinically relevant SNVs/indels for nine (3.9%) of 233 individuals with no previously identified pathogenic CNVs (Fig. 1).

**Table 1:** Clinically relevant SNVs and indels in NDD-genes identified in eleven of 259 adults with schizophrenia.

Case	Sex	AAO (y)	FHx	ID/ syndromic	Rare CNV group	Clinically relevant CNV	Gene (transcript)	OMIM ID	Variant type	Variant	Chromosomal position (GRCh37/hg19)	Allele frequency (ExAC/gnomAD)	Validation	Gene function
591	F	20	No	No/No	No	No	*KCNQ5* (NM_001160130.1)	607357	Stopgain	c.C1984T: p.(Q662*)	chr6:73904349C>T	0/0	NA	Synaptic transmission
367	F	52	No	No/No	No	No	*SCN8A* (NM_001177984.2)	600702	Deletion (frameshift)	c.1940_1957delins: p.(G647Vfs*18)	chr12:52115634 GCGTGGTGTCCCTCATCG>GT	0/0	NA	
592	M	17	No	Mild/Yes	No	No	*CACNA1A* (NM_001127221.1)	601011	Deletion (frameshift)	c.2042_2043del: p.(Q681Rfs*100)	chr19:13414644 CCT>C	0/4.06 × 10^−6^	NA	
567	F	NA	Yes	Mild/No	No	No	*SHANK3* (NM_033517.1)	606230	Deletion (frameshift)	c.2183_2184del: p.(Q728Qfs*12)	chr22:51153434 CAG>C	0/0	NA	
28	M	20	Yes	No/No	Yes^VUS^	No	*RYR2* (NM_001035.2)	180902	Missense	c.C13489T: p.(R4497C)	chr1:237954741C>T	0/0	NA	
56	F	16	No	Mild/Yes	Yes	16p11.2 microduplication	*SCN1B* (NM_001037.4)	600235	Missense	c.C363G: p.(C121W)	chr19:35524558C>G	8.24 × 10^−6^ /4.06 × 10^−6^	Sanger^mat^	
55	F	31	Yes	Mild/Yes	Yes	16p11.2 microduplication	*SYN1* (NM_133499.2)	313440	Missense	c.G1648A: p.(A550T)	chrX:47433735C>T	0/0	Sanger	
573	M	44	No	Mild/No	Yes^VUS^	No	*MEIS2* (NM_001220482.1)	601740	Stopgain	c.C1099T: p.(Q367*)	chr15:37187379G>A	0/0	NA	Regulation of gene expression
625	F	24	No	Borderline/Yes	No	No	*BRPF1* (NM_001003694.1)	602410	Stopgain	c.C3346T: p.(R1116*)	chr3:9788005C>T	0/0	Sanger	
609	M	24	No	Borderline/Yes	No	No	*BRPF1* (NM_001003694.1)	602410	Insertion (frameshift)	c.2228dupA: p.(E743fs*5)	chr3:9784853G>GA	0/0	WES, DNV	
92	F	29	Yes	No/No	Yes^VUS^	No	*MECP2* (NM_004992.3)	300005	Deletion (frameshift)	c.1157_1163del: p.(L386Hfs*21)	chrX:153296115 TGGGGGCA>T	0/0	Sanger	

Rare SNVs and indels considered clinically relevant, together with their key genetic parameters are shown. Two of these individuals also have clinically relevant CNVs (16p11.2 microduplications)

*F* female*, M* male*, AAO* age at onset, *y* years, *NA* not available, *FHx* family history of schizophrenia/psychosis, *ID* borderline to mild intellectual disability, *CNV* copy number variant, *VUS* CNV of uncertain clinical significance, *OMIM* online mendelian inheritance in man, *mat* maternally inherited, *WES* whole-exome sequencing, *DNV* de novo variant. See Supplementary Table S3 for VUS CNVs.

#### Rare disease-associated tandem repeat loci are expanded in schizophrenia

In three individuals, we identified and validated CTG TREs involving two of the 45 disease-associated tandem DNA repeats assessed. Two individuals had potentially damaging TREs in *ATXN8OS* and one had a potentially pathogenic TRE in *DMPK* (Table 2, Supplementary Fig. S2). The expanded CTG repeat (>200 repeats) at the 3’ untranslated region (UTR) of *DMPK* was paternally inherited, with evidence of typical variable expression of the associated condition, myotonic dystrophy type 1 (DM1)^42^ (Table 2). Both expanded (>200 repeats) CTG repeats at the 3’ UTR of *ATXN8OS* were found to be maternally inherited/derived (Table 2); there was no clinical or family history of typical neuromuscular features of the associated spinocerebellar ataxia type 8 (SCA8) disorder^43^.

**Table 2:** Three individuals with schizophrenia and TREs identified in the 3’UTR of genes *DMPK* and *ATXN8OS*.

Gene	OMIM ID Disease	Case ID /family members	Sex	AAO (y)	FHx of SCZ	FHx of OMIM disease	ID	Other features	Rare CNV	CTG repeat				
										Chromosomal position (GRCh37/hg19)	Size			
											Normal	Pathogenic	EH-estimated	Validated
*DMPK*	160900 DM1	Proband 72	M	19	No		No	Episodes of severe constipation	Yes^VUS^	chr19:46273174 -46273699	5–35	≥51	97	>200
		Father	M	NA	NA	Yes	No	Cataract	NA				NA	>200
		Mother	F	NA	NA		No	No	NA				NA	~9
*ATXN8OS*	603680 SCA8	Proband 329	M	21	Yes^a^		No	No	No	chr13:70713162 -70713778	15-44	>110	116	>200
		Father	M	NA	NA		No	No	NA				NA	~20
		Mother	F	NA	NA	No	No	Unilateral “glaucoma”	NA				NA	>200
		Proband 11	F	17	No		Yes	Cataracts	Yes^VUS^				91	>200
		Brother	M	NA	NA		NA	No	NA				NA	~20
		Mother	F	NA	NA	No	Yes	No	Yes^VUS^				NA	~100

TREs identified in *DMPK* and *ATXN8OS* in three individuals with schizophrenia and their family members, together with their key clinical and genetic variables are shown

*F* female*, M* male*, AAO* age at onset, *y* years, *NA* not applicable, *FHx* family history, *SCZ* schizophrenia/psychosis, *ID* borderline to mild intellectual disability, *CNV* copy number variant, *OMIM* online Mendelian inheritance in man *EH* ExpansionHunter; See Supplementary Table S4 for a list of tandem repeat loci examined in this study

^a^Proband 329 has a brother with schizophrenia; VUS, CNV of uncertain clinical significance, e.g., both proband 11 and her mother are identified to have a ~790 Kb deletion of uncertain significance overlapping *ZNF804A*; see Supplementary Table S3 for all VUS CNVs

Therefore, of the 233 individuals with no pathogenic CNVs, we propose 14 individuals (6%) with rare high-impact variants (i.e., clinically relevant SNVs/indels, or TREs with clinical implications) (Fig. 1).

#### Females and individuals with learning/intellectual disabilities may have enhanced clinical yield from genome sequencing in schizophrenia

**Fig. 2:**
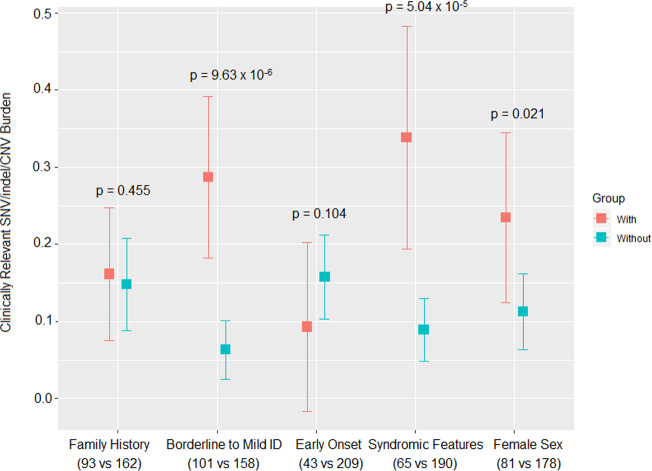
Genetic risk for schizophrenia and clinical/demographic variables. This figure shows results for analyses of five clinical variables/features (family history of schizophrenia/psychosis, ID, early age at onset, mild syndromic features, and biological sex) with respect to rare clinically relevant variant burden, defined as the number of CNVs and/or SNVs/indels per individual. Orange and blue coloured boxes, and vertical bars representing 95% confidence intervals, indicate respectively results for individuals with and without each of the five variables; numbers for each subgroup are indicated in brackets under variable labels (Supplemental Table S5), and *p*-values for analyses are provided above graphed results. Clinically relevant rare variant burden was significantly greater for females, individuals with broadly defined ID, or with mild syndromic features.

Individuals with learning and intellectual disabilities, as expected from previous studies of this cohort and other studies^16,17,44^, were significantly enriched for rare clinically relevant CNV and/or SNV/indel variants (*p* = 9.63×10^−6^, Fig. 2). Results for clinically relevant variants were also significant for individuals with syndromic features (*p* = 5.04×10^−5^), and female sex (*p* = 0.021), but not for family history or age at onset (Fig. 2). Notably however, six (3.8%) of 158 individuals with no learning or intellectual disabilities (Supplementary Table S5) had a clinically relevant SNV/indel or disease-associated CTG TRE.

### Additional findings relevant to the broader genetic architecture of schizophrenia

#### Rare clinically relevant SNVs/indels disrupt genes associated with neurodevelopmental pathways

The SNVs and indels identified to be of potential clinical relevance involve 10 genes associated with synaptic transmission (*n* = 7) or chromatin remodelling and transcription regulation (*n* = 3) (Table 1), consistent with pathways previously implicated in NDDs, including schizophrenia (Methods). Of the seven synaptic transmission genes, four harboured variants in genes encoding components of voltage-gated ion channels: *KCNQ5* p.(Q662*), *CACNA1A* p.(Q681Rfs*100), *SNC8A* p.(G647Vfs*18), and *SCN1B* p.(C121W), involving 1.5% (*n* = 4) of the 259 individuals studied (Table 1). Of the genes involved in the regulation of gene expression, in two unrelated individuals, both with borderline ID and mild syndromic features^31^, we identified distinct ultra-rare (i.e., not seen in the general population) LoF variants affecting exons 13 and 7 of *BRPF1*, respectively a nonsense p.(R1116*) variant validated by Sanger sequencing and a confirmed de novo frameshift p.(E743fs*5) variant (Table 1, Supplementary Fig. S3).

#### Evidence for *ZMYM2* as a novel schizophrenia-candidate gene

In addition to *BRPF1*, we identified three other LoF-intolerant genes with multiple deleterious ultra-rare SNVs/indels in the schizophrenia cohort studied, and compared results to findings from previous studies of schizophrenia and other disorders (Table 3). The top candidate gene identified was *ZMYM2* with three rare LoF variants in three unrelated individuals (*p* = 9.51×10^−6^); one had a *NRXN1* deletion (Fig. 1, Table 3, Supplementary Table S2). *ZMYM2* was supported by substantial evidence from the literature^45–47^ including suggestive meta-analysis results from exome studies of schizophrenia (SCHEMA *p*-value = 1.79 × 10^−5^)^40^, and no rare LoF variants detected in our TOF-control sample (Table 3, Supplementary Fig. S4). Supplementary Table S8 shows the LoF and rare missense variants in *ZMYM2* identified in our cohort and reported by others. Though interesting genes with evidence of greater constraint, i.e., lower gnomAD LoF observed/expected upper bound fraction (LOEUF), variant results for *GPRIN1* and *DNAJC6* were less compelling as candidate genes of potential clinical relevance (Table 3).

**Table 3:** Genes with deleterious ultra-rare LoF variants identified in two or more individuals with schizophrenia in the sample studied.

Gene (gnomAD LOEUF)	This schizophrenia sample (total *n* = 259)						Results from other studies			Gene function
	Number of individuals with variants in gene	Variant	Case	Other clinically relevant variant	Binomial *p*-value	FDR	Number of LoF variants^a^	References	SCHEMA meta-analysis *p*-value^b^	
*ZMYM2* (0.33)	3	c.G1960T: p.(E654*)	562^rare^	2p16.3 *NRXN1* deletion	9.51×10^−6^	0.028	7 SCZ, 2 ASD, 7 ID/DD	^45–47,57^	1.79 × 10^−5^	Regulation of gene expression
		c.2253dupT: p.(S751Rfs*2)	183^rare^	No						
		c.763_766del: p.(T343Lfs*37)	291	No						
*GPRIN1* (0.32)	2	c.2600_2604del: p.(E867Afs*40)	34^rare^	No	9.08×10^−5^	0.134	1 SCZ	^45^	1	Synaptic development
		c.1334_1335del: p.(V445Gfs*27)	16^rare^	No						
*BRPF1* (0.18) (also in Table 1)	2	c.C3346T: p.(R1116*)	625	No	3.67×10^−4^	0.360	3 SCZ	^6,45^	0.017	Regulation of gene expression
		c.2228dupA: p.(E743fs*5)	609^c^	No						
*DNAJC6* (0.22)	2	c.C988T: p.(R330*)	585	No	7.39×10^−4^	0.544	0	**_**	0.527	Vesicle mediated transport
		c.94_95del: p.(S32Rfs*49)	26	No						

Binomial *p*-value and FDR were calculated as described in Supplementary Information. Ultra-rare: not seen in the general population

*LoF* loss of function, *FDR* false discovery rate, *gnomAD* genome aggregation database, *LOEUF* loss-of-function observed/expected upper bound fraction, *SCZ* schizophrenia, *ASD* autism spectrum disorder, *ID/DD* intellectual disability/developmental delay

^a^The number of previously reported LoF variants in NDDs is indicated for each gene and condition associated. See Supplementary Table S8 and Supplementary Fig. S4 for details of *ZMYM2* variants in this and other cohorts. Superscript ^rare^ indicates an individual belonging to the rare CNV subgroup (see Supplementary Table S3)

^b^Genome-wide *p*-value reported in SCHEMA exome-sequencing meta-analysis (schema.broadinstitute.org/); only the p value for *ZMYM2* met criteria for genome-wide suggestive

^c^In this individual, the LoF variant in *BRPF1* was confirmed as a de novo variant (see Table 1, Supplementary Fig. S3)

#### Contribution of other rare exonic variants and polygenic risk

Using our WGS data we examined the ten genes showing genome-wide significant association with schizophrenia on meta-analyses using SCHEMA exome sequencing data^40^. In these ten genes we detected between one and 12 (in *SETD1A*) rare missense exonic variants, none of which were considered clinically relevant (Supplementary Table S9).

Analyses using common variants showed that our schizophrenia cohort had a significantly higher mean PRS compared to the TOF-control group, explaining 9.5% of the variance (Nagelkerke’s pseudo *R*^2^ from logistic regression at *P*_T_ = 0.05, *p* = 1.07×10^−9^) (Supplementary Fig. S5). Mean PRS was not significantly different between those with and without clinically relevant rare variants (p = 0.52) (Supplementary Fig. S6a). Sixteen (6.23%) individuals with schizophrenia (Fig. 1), fell in the top twentieth PRS percentile subgroup (i.e., where the odds ratio (OR) was greatest relative to the remainder of the sample, OR = 2.92, 95% confidence interval: 1.05–8.11) (Supplementary Fig. S7). Individuals with a positive family history of schizophrenia/psychoses showed a higher mean PRS than those without such a family history (*p* = 0.046); no other results for clinical/demographic variables achieved significance though there was a non-significant trend for higher mean PRS in individuals with no learning and/or intellectual disabilities (*p* = 0.085) (Supplementary Fig. 6b).

Per our original study design^16,17^, we also compared findings between the 136 individuals with rare CNVs, including those of uncertain clinical significance (all identified by the WGS pipeline; Supplementary Tables S2, S3), to the 123 individuals with no rare CNVs. There were no significant differences, respectively, for the 11 rare SNVs/indels (*n* = 5 vs *n* = 6, Fisher’s exact test, *p* = 0.76), global burden of ultra-rare LoF variants (one-sided Wilcoxon signed-rank test, *p* = 0.0975, data not shown), or mean PRS (*p* = 0.37) (Supplementary Fig. 6a).

## Discussion

Using genome sequences from 259 unrelated schizophrenia-affected adults, and simultaneously interrogating for a range of genetic variants, we undertook a conservative approach to explore the clinical relevance of other types of variants to schizophrenia beyond copy number variation. We considered only rare SNVs and indels with a strong association with NDDs and determined that about 4% of the studied individuals had such clinically relevant, predominantly LoF, variants. In addition, we identified trinucleotide TREs with potential clinical implications in other individuals. The fact that 14 (5.4%) of 259 individuals with schizophrenia, including many with no broadly defined learning disability/ID, had a high-impact SNV/TRE not detectable by CMA, provides an initial indication that an important minority of patients in the community would be found to have such clinically relevant variants using WGS. Notably, several individuals had multiple genetic risk factors (Fig. 1, Table 1), consistent with polygenicity within individuals, and reduced penetrance, even of high-impact clinically relevant variants in schizophrenia^17,48,49^.

A novel finding was the identification of individuals with schizophrenia and TREs associated with DM1 and SCA8, neuromuscular disorders with highly variable expressivity and neuropsychiatric manifestations^15,42,43^. A recent genome-wide study of >17,200 individuals identified rare CTG TREs in *DMPK* in individuals with ASD^50^, and older studies reported high prevalence of psychotic disorders in individuals with TREs in *DMPK* and juvenile DM1^51^. Older technologies had provided initial evidence linking CTG repeats in *ATXN80* and SCA8 to major psychoses^15^, and overall TRE results may be to some extent consistent with other historical studies^14,52–54^. With continuous technical improvements in genome-wide detection of such expanded repeats and more precise size estimations, we expect to identify other, novel unstable TREs in individuals with schizophrenia, and elucidate the underlying mechanisms that lead to psychiatric expression.

The fact that the clinically relevant rare SNVs/indels identified involved genes implicated in synaptic transmission and transcription regulation pathways previously associated with neurodevelopment and schizophrenia^6,8,37^, further illustrates the potential for WGS to contribute to etiological understanding with convergence on mechanisms of importance to schizophrenia pathogenesis. In an iterative fashion, each genome can potentially inform future adjudication of other clinically relevant variants, and can be reanalyzed as further discoveries are made^55^.

The clinical/demographic findings suggest that not only individuals with any degree of intellectual impairment but also female patients may disproportionately benefit from clinical genetic testing in schizophrenia^16^. The latter finding is consistent with the possibility that there may be in the general population a female protective mechanism for schizophrenia analogous to that proposed in other NDDs with male bias of expression, such as ASD^56^. Studies of larger schizophrenia cohorts are needed to investigate this phenomenon.

In addition to findings of possible immediate impact to clinical translation, the sequencing data allowed us to examine several other pertinent aspects of genetic architecture in schizophrenia. This includes our proposal of Zinc Finger MYM-Type Containing 2 (*ZMYM2*) (OMIM: 602221) as a putative schizophrenia-risk candidate gene of possible clinical relevance, supported by a gene-based analysis of deleterious ultra-rare variants in LoF-intolerant genes, SCHEMA meta-analysis data^40^, and LoF variants in *ZMYM2* reported in other studies examining NDDs, including ASD, ID and schizophrenia^45–47,57^. Comparable to other schizophrenia-associated genes, variants in *ZMYM2* appear to manifest pleiotropic effects^45–47,57^. *ZMYM2* encodes a zinc finger protein, which may act as a transcription factor and thereby regulate gene expression (Supplementary Fig. S4), a mechanism elucidated for other clinically relevant variants (Table 1). Further studies examining the function of the encoded protein during neurodevelopment, and analysis of larger psychiatric cohorts, are required to establish a robust link between LoF variants in *ZMYM2* and schizophrenia pathogenesis. Functional studies and further sequencing data will also be needed for more broadly defined rare nonsynonymous variants, given their expected lower impact than ultra-rare LoF variants. This would include results for genes proposed through exome-sequencing and meta-analysis (e.g., SCHEMA consortium; Supplementary Table S9)^40^, in order to determine their clinical relevance and contribution to overall schizophrenia liability.

We also took advantage of WGS data to simultaneously assess the potential contribution of common variant burden (e.g., explaining an estimated 9.5% of the variance using PRS data) and rare clinically relevant variants, allowing for examination of a broader-based risk profile for each individual, consistent with the proposed polygenic nature of schizophrenia. Unlike previous studies that used imputed genotyping data to study PRS^58^, here we implemented precisely genotyped SNP data as determined by WGS (not possible using WES or CMA). We did not identify a correlation between PRS and the burden of rare, clinically relevant variants (Supplementary Fig. S6a)^18^. While this may in part have been affected by the exclusion from this cohort of individuals with one of the highest known risks for schizophrenia, 22q11.2 deletions, there are as yet limited data on PRS in the context of other high-impact variants associated with schizophrenia^18^. Consistent with other studies^58^, individuals with a family history of schizophrenia/psychosis showed some enrichment for schizophrenia PRS, (supplementary information and Supplementary Fig. S6b), but limited availability of parental DNA samples precluded variant segregation analyses to confirm the transmission of SNP-based risk alleles. Judging by the modest estimated risk conveyed by the highest PRS (OR = 2.92; 95% CI: 1.05-8.11), and consistent with other reports^59^, PRS is not yet sufficient to apply clinically for individual schizophrenia risk classification. Nevertheless, the growing numbers of individuals with available WGS data deserve further study of the potential clinical application of polygenic risk prediction, and effects on this of high-impact rare variants^18,60^.

Our results should be interpreted in the context of a few important limitations. First, by design, and comparable to a companion study of TOF^19^, about half of this cohort had rare genic CNVs, as determined by previous CMA analysis^16,17^. The majority of such CNVs are not clinically pathogenic (Supplementary Tables S2, S3), nevertheless, this may have influenced our findings, including the clinical yield of SNVs, TREs and SVs, and results for clinical variables. Analysis of larger cohorts of schizophrenia with and without clinically relevant CNVs would be needed to more precisely estimate their impact relative to other high-impact variants and polygenic risk, and relationships to clinical phenotypes^18,61^. Second, due to technical limitations and the complex nature of tandem repeats, we could not determine the precise size of TREs^39^. Size underestimation may have hindered the detection of other tandem repeats contributing to schizophrenia risk in this cohort. Third, inability to identify schizophrenia-relevant LoF SVs may have been in part due to the limited resources currently available for the interpretation of such variants. Efforts are underway to construct comprehensive resources for SVs, which together with the improvements in our understanding of the complex etiology and genetic mechanisms of schizophrenia will enhance the identification of phenotypically relevant variants through WGS-based approaches. Fourth, using a cohort of 225 adults with congenital cardiac disease as controls might have produced more conservative results for our analyses than if using other control groups without a developmental, albeit cardiac, phenotype. However, there are few known links between genetic risk for TOF and for schizophrenia (apart from e.g., 22q11.2 deletions, 1q21.1 duplications, and deleterious variants in *RYR2* (Supplementary information)). Fifth, our clinical adjudication of variants could also be considered overly conservative, relying on currently available results for NDD, and 45 established genes for TREs. Future efforts to refine clinical interpretation of rare variants for schizophrenia will be essential.

In conclusion, our results provide important evidence of the enhanced performance of WGS compared to CMA in the detection of genome-wide clinically relevant variants^62^, and an initial indication of features that could help identify individuals with schizophrenia who are most likely to benefit from clinical genetic testing and genetic counselling^16,17^. The results also reiterate the complexity and pleiotropy of schizophrenia, and suggest the interplay of multiple variant types, each with varied expressivity and penetrance, in every individual. With continued improvements in high-throughput sequencing technologies, WGS will become more affordable, which together with advances in interpretation (particularly for variants affecting non-coding and regulatory elements) promise to make WGS an ideal tool for routine diagnostic practice^63^. Global efforts combining WGS data from various neuropsychiatric disorders will shed light on the shared and disparate genetic factors and mechanisms underlying these disorders^50^. Eventually, implementation of clinical WGS will extend to patients with schizophrenia, as for those with other NDDs, to further guide our understanding of prognosis, medical management, and familial recurrence risk assessment, and as part of global efforts towards “precision medicine”.

## Supplementary information

Supplementary tables

Supplementary methods and figures
