# Supplementary material for: Genome sequencing broadens the range of contributing variants with clinical implications in schizophrenia
Source: Transl Psychiatry. 2021 Feb 1;11:84. doi: 10.1038/s41398-021-01211-2 (PMC7851385; doi:10.1038/s41398-021-01211-2)
Supplement: Supplementary file 2 — Supplementary methods and figures [file 41398_2021_1211_MOESM2_ESM.pdf]

Supplementary information for "Genome sequencing broadens the range of contributing variants with clinical implications in schizophrenia."

Methods:

Whole genome sequencing (WGS) and variant quality control: Genomic DNA was extracted from blood (n=227) or saliva (n=32) (**Supplementary Table S6**). All 259 DNA samples were assessed for quality, and DNA library preparation was performed PCR-based (n=252) and PCR-free (n=7). All samples were sequenced using the Illumina HiSeq X platform at The Centre for Applied Genomics (TCAG, Toronto, Canada) as previously described.[1,2]

Alignment and variant calling (single nucleotide variants (SNVs), small insertions and deletions (indels), structural variants (SVs), copy number variants (CNVs)): Reads were aligned to the reference genome (build GRCh37/hg19) using the Burrows-Wheeler Aligner version 0.7.12 as a sorted binary alignment map (BAM) format. Duplicate reads were removed by MarkDuplicates from Picard version 2.5.0. Genome Analysis ToolKit (GATK) version 3.7.0 was used for indel realignment, quality score recalibration and SNV and indel calling, followed by their annotation using ANNOVAR. For the identification of structural variants (SVs), we implemented two algorithms commonly used for SV detection: LUMPY and MANTA.[3,4] For SV analysis, we focused on variants smaller than 10Kb, which are not reliably detectable using CMA. CNVs (≥10Kb) were called using ERDS and CNVnator; we only included variants called by both algorithms for further analysis.[5] Read alignments for putative SNVs, indels, and the presence of SVs and CNVs, were manually inspected from the BAM using IGV.

Assessment of the pathogenicity of rare variants (SNVs, indels, SVs, CNVs): All rare (defined as population allele frequency ≤0.01) exonic and exonic-splicing SNVs and indels, SVs, and CNVs were analyzed for their potential pathogenicity. Population maximum allele frequency of each variant was

derived from data included in ExAC, 1000 Genomes Project, and gnomAD and gnomAD SV databases.[6-9] Probability of loss-of-function (LoF) intolerance was measured by the upper bound of a Poisson-derived confidence interval around the ratio of the observed/expected number of LoF variants in every gene were derived from gnomAD (v2.1.1), represented by loss-of-function observed/expected upper bound fraction (LOEUF) score.[6] LoF variants were defined as stop-gains, frameshift indels, and splice-site variants. Rare nonsynonymous variants with high predicted scores in 5 of 8 commonly used *in silico* algorithms: CADD (≥15), SIFT (≤0.05), PolyPhen2 HVAR (≥0.90), Provean (<-2.5), ma (≥1.90) and mt (≥0.5) scores, PhyloPMam (≥2.30) and PhyloPVert (≥4.0) were considered as deleterious and were further assessed for pathogenicity.[10] Given the evidence for genetic overlap between schizophrenia and other major neurodevelopmental disorders (NDDs), we considered loci and genes as potentially associated with schizophrenia if they had been implicated in any NDD (such as intellectual disability (ID) or autism spectrum disorder (ASD)), and their implicated pathways.[11-26] Rare small variants were classified with respect to pathogenicity according to guidelines provided by the American College of Medical Genetics and Genomics, into five standard categories for SNVs and indels: (1) pathogenic, (2) likely pathogenic, (3) variant of unknown significance, (4) likely benign and (5) benign, and five standard categories for CNVs: (1) pathogenic, (2) uncertain clinical significance, likely pathogenic, (3) uncertain clinical significance, likely benign, (4) uncertain clinical significance (no subclassification), and (5) benign.[10,27-29] Pathogenicity of rare SVs was assessed using their predicted damaging or deleterious effects on genes implicated in NDDs. In this study, for CNVs, SNVs and indels, we considered pathogenic and likely pathogenic variants as potentially clinically relevant and contributing to the expression of schizophrenia.

Independent confirmation of SNVs/indels: To confirm the WGS-based detection of SNVs and indels of interest and determine their inheritance, we PCR amplified DNA from participants along with that available from their family members. This was followed by Sanger sequencing in four cases (625, 56, 55 and 92). For one case (609), we assessed WES trio data available for the proband and both parents. DNA

was only available for one other complete trio (case 56) for variant confirmation and segregation analysis (**Supplementary Figure S3**).

Statistics: Statistical analyses were conducted using R version 3.3.2 software (The R Foundation). We hypothesized that individuals with ID, syndromic features, female sex, younger age at onset, and positive family history of schizophrenia /psychosis would have a greater contribution from the specific groups of rare and common genetic variants under investigation.[28,30-33] Therefore, all p values presented, unless stated otherwise, were calculated using one-sided Wilcoxon signed-rank test.

Polygenic risk quantification: Comparable WGS data from 225 individuals with tetralogy of Fallot (TOF) and related congenital cardiac disease were used as non-psychiatric control group for schizophrenia polygenic risk score (PRS) analysis. TOF and schizophrenia share only a few genetic susceptibility loci.[24,34-36]

Identity-by-descent (IBD) proportions were estimated by the genome option of PLINK (version 1.9) to ensure that individuals from the schizophrenia cohort, as well as those in the TOF cohort were not related and did not otherwise overlap. Using principal component analysis (PCA) and SNP data using 1000 genomes database as reference ancestry groups, we inferred ancestry and examined for population stratification in schizophrenia-cases and TOF-controls (**Supplementary Figure S1**). Based on these results, data for two outlying individuals (367 with a rare SNV of *SCN8A*, and 574 with a rare X chromosome CNV) from the schizophrenia cohort (**Table 1** and **Supplementary Table S3)** were excluded prior to PRS analysis.

PRS for each schizophrenia-case and TOF-control individual was calculated by summing the number of risk alleles weighted by the SNP's log odds ratio as described previously.[37,38] The application of PLINK toolset (version 1.9), and built-in filtering steps from PRSice (v1.25) (the tool commonly used for the calculation of PRS) were employed for quality control steps (implemented in R version 3.3.2). High-

quality SNPs were mapped to hg19 coordinates to select for the SNPs that intersected with our WGS data. Briefly, SNPs with minor allele frequency (MAF) <1%, missingness rate >1%, and Hardy-Weinberg equilibrium exact test p value $<1 \times 10^{-5}$ were excluded.

The proportion of case-control variance explained (Nagelkerke's R^2) was calculated by PRSice software[38] across a range of training data p value thresholds (P_T) (**Supplementary Figure S5**); the top ten PCs were included in the final model as covariates to adjust for potential population stratification. All primary PRS results presented here were generated using a P_T of 0.05, as this threshold explained the most case-control variance in the 2014 PGC schizophrenia meta-analysis.[37] At this threshold, a total of 58,359 individual SNPs meeting quality control criteria from the training dataset were mapped in our samples.

Independent confirmation of tandem repeat expansions (TREs): We experimentally validated the presence of selected TREs identified by ExpansionHunter[39] in individuals with schizophrenia to exclude any false discoveries. TREs identified in *DMPK* and *ATXN8OS* were validated using repeat-primed PCR (RP-PCR) followed by fragment analysis with FAM-labelled primers and capillary electrophoresis using the following primers:

Primer tail sequence: 5'- TACGCATCCCAGTTTGAGACGC-3'

CTG-repeat-binding primer: 5'- TACGCATCCCAGTTTGAGACGC AGCAGCAGCAGCAGCA-3'

DMPK-specific primer: 5'- FAM-CGAACGGGGCTCGAAGGGTCCTTGT-3'

ATAXN8OS-specific primer[40]: 5'- FAM-CTGGGTCCTTCATGTTAGAAAACCT-3'

Recurrent LoF variant burden analysis: Inspired by results of previous studies,[41,42] we hypothesized that a higher burden of LoF variants in schizophrenia individuals compared to the general population (using ExAC, gnomAD, and 1000 genomes databases[6-8]) would be associated with an increased schizophrenia risk under a haploinsufficiency model. We examined the overall burden of disruptive ultra-rare variants

(dURVs) in schizophrenia individuals, i.e., variants that are not seen in the general population and were observed only once in our schizophrenia cohort.

For single genes with LOEUF score<0.35,[6] we tested whether the overall burden of dURVs was significantly greater than expected adapting the *de novo* burden test based on background mutation rate implemented in DenovolyzeR.[43] Notably, this method does not require matched controls. Since the original background mutation rates were estimated for *de novo* variants, we applied a global scaling factor computed so that the number of predicted and observed dURVs match. This global factor was formulated as follows:

$$global\ scaling\ factor = \frac{number\ of\ observed\ dURVs}{number\ of\ samples\ \times\ \sum P(expected)\ de\ novo\ dURVs\ for\ all\ genes}$$

The adjusted *de novo* mutation probabilities were used to test the dURV burden significance for each gene using a binomial test. The Benjamini-Hochberg method was applied to adjust for multiple comparisons.

Supplementary Figures:

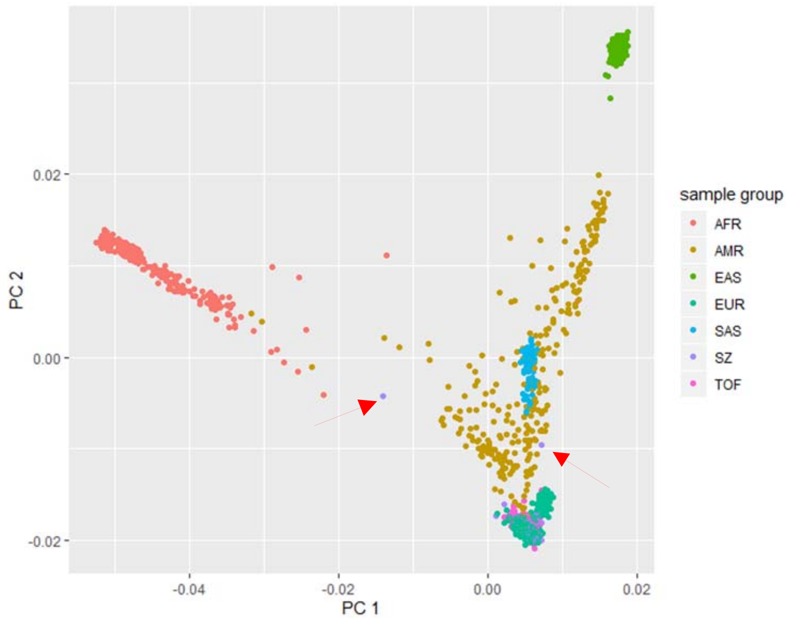

Figure S1. Sample ancestry analysis. Principal component analysis for individuals in the schizophrenia (n=259) and TOF (n=225) cohorts using African (AFR), mixed American (AMR), East Asian (EAS), South Asian (SAS), and European (EUR) samples from the 1000 Genomes database (https://www.internationalgenome.org/). Two outlier schizophrenia individuals (marked with red arrowheads) were excluded from PRS analysis.

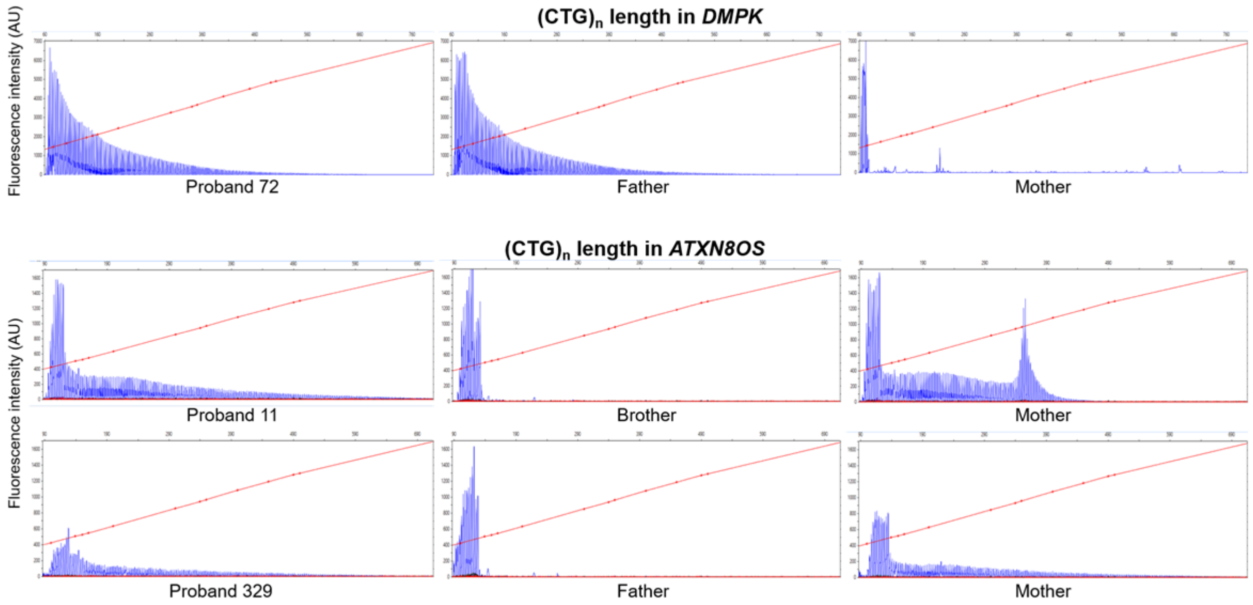

Figure S2. Determining the size of CTG repeats in *DMPK* and *ATXN8OS*. The figure shows results of repeat-primed PCR validation studies of long CTG repeats that had been predicted using ExpansionHunter for three probands with schizophrenia. Number of CTG repeats were estimated based on the length of the CTG tract quantified by fluorescence intensity (arbitrary units). The top panel shows expanded CTG tandem repeats (>200) in the 3'UTR of *DMPK* in proband 72 and the paternal inheritance. The middle and bottom panels show results for CTG repeats in *ATXN8OS*. For proband 11 (middle panel), there were >200 repeats, and the expansion was possibly inherited from an allele of the mother's with a relatively shorter premutation CTG repeat tract (~100 repeats); the results showed no expansion for the proband's brother. For proband 329 (bottom panel) there is an expansion of CTG tract (>200 repeats) with maternal inheritance.

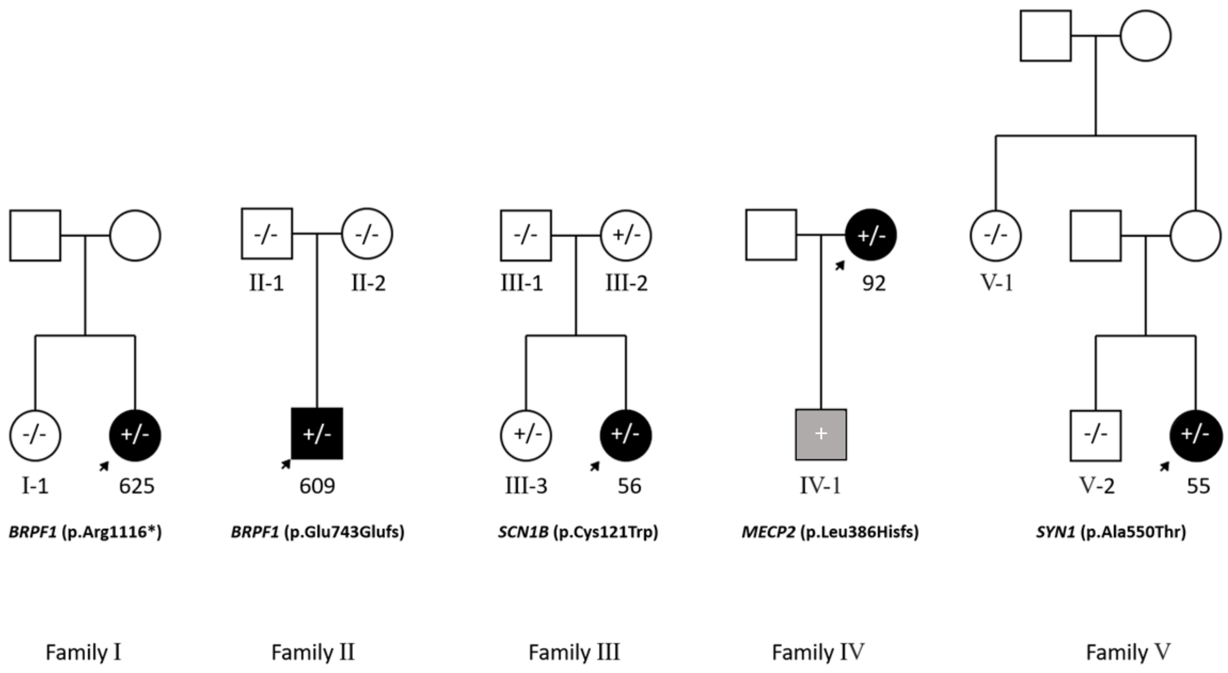

Figure S3. Pedigrees (I-V) of five individuals with schizophrenia. Five of eleven schizophrenia-affected individuals (indicated with arrows) identified to have a putatively clinically relevant SNV/indel and their family members are depicted. In each pedigree, black and grey-shaded individuals are affected with schizophrenia and psychotic mood disorder, respectively. DNA was collected with informed consent from individuals with a number underneath, and Sanger sequencing was performed to confirm the presence or absence of the variant of interest. For case 609 (Family II), inheritance was determined using available WES data. Confirmed genotypes (+ = with; - = without) for each variant indicated below the pedigree are shown for individuals tested.

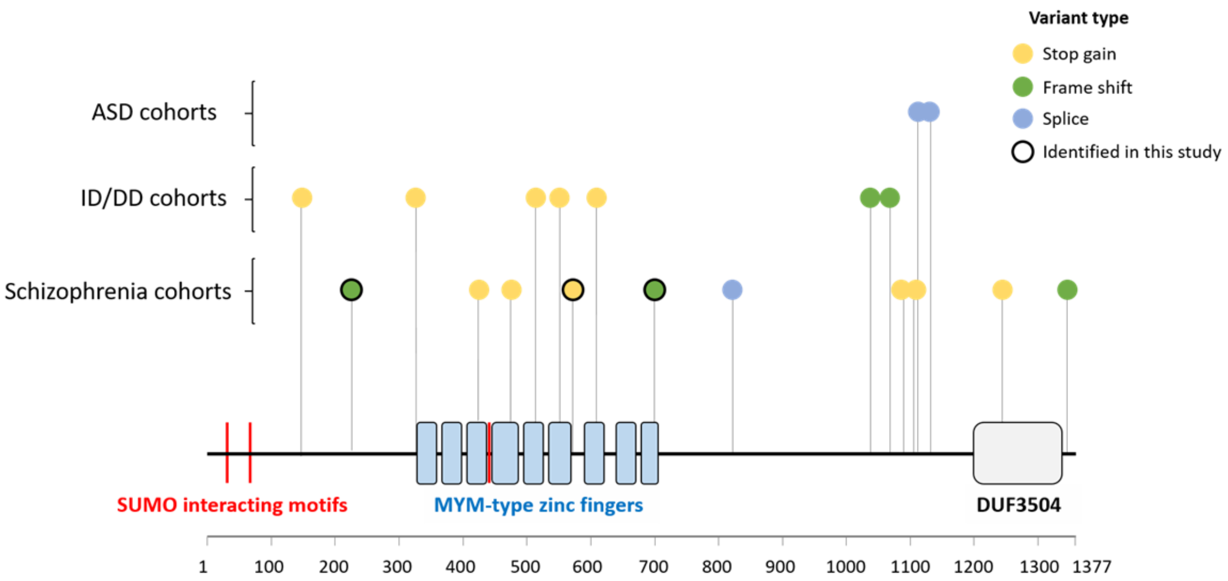

Figure S4. Schematic of ZMYM2 protein domains with identified LoF variants in NDDs. *ZMYM2* encodes a zinc finger protein, which may act as a transcription factor and thereby regulate gene expression.[44-47] ZMYM2 protein domains with the position of LoF variants shown in schizophrenia (n=10) and related NDD disorders (n=9) (see **Supplementary Table S8** for details of variants and studies). Stop gain, frame shift and missense variants are shown with yellow, green and blue circles, respectively. Variants identified in this study of schizophrenia are indicated with black outlined circles. Vertical red lines indicate small ubiquitin-like modifier (SUMO) interacting motifs, blue rectangles indicate conserved MYM-type zinc finger motifs, and a large grey rectangle indicates the C-terminal 313-amino acid DUF3504 domain of unidentified function. Formerly, the 27-exon gene *ZMYM2* encoding the ZMYM2 protein was known as *ZNF198*.

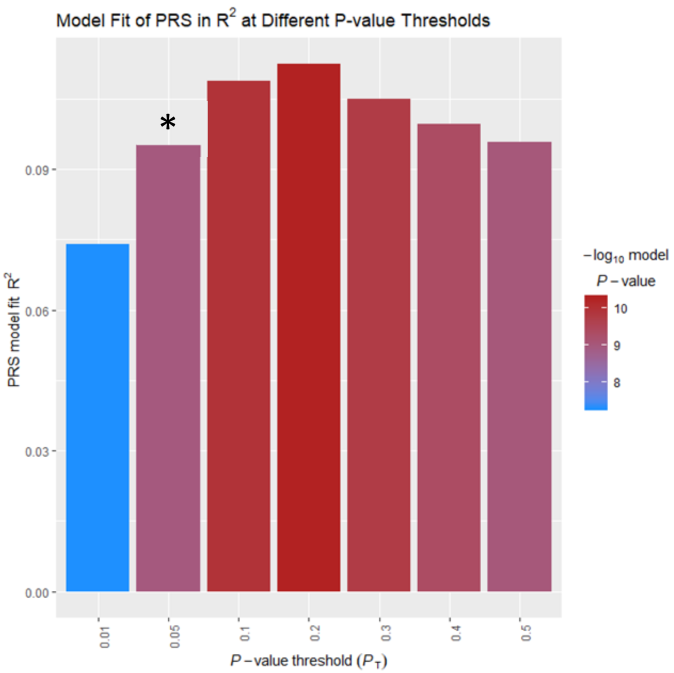

Figure S5. Polygenic risk R^2 calculated at different P-value thresholds (P_T). Y-axis shows Nagelkerke's R^2 at each P_T. We observed the greatest predictive ability for a score constructed of 143750 SNPs with P-value threshold 0.2 using the PGC2 data.[37] Results for P_T = 0.05 (marked with an asterisk on the plot), comprising 58,359 SNPs, were used for all analyses in this study, as suggested by results from the second Psychiatric Genomics Consortium mega-analysis of schizophrenia (PGC2 schizophrenia).[37]

a

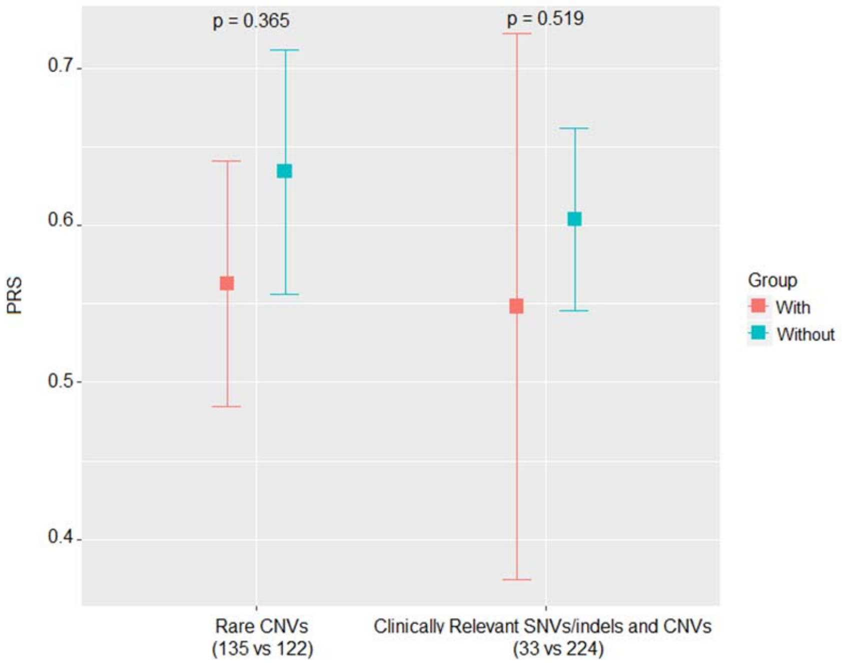

b

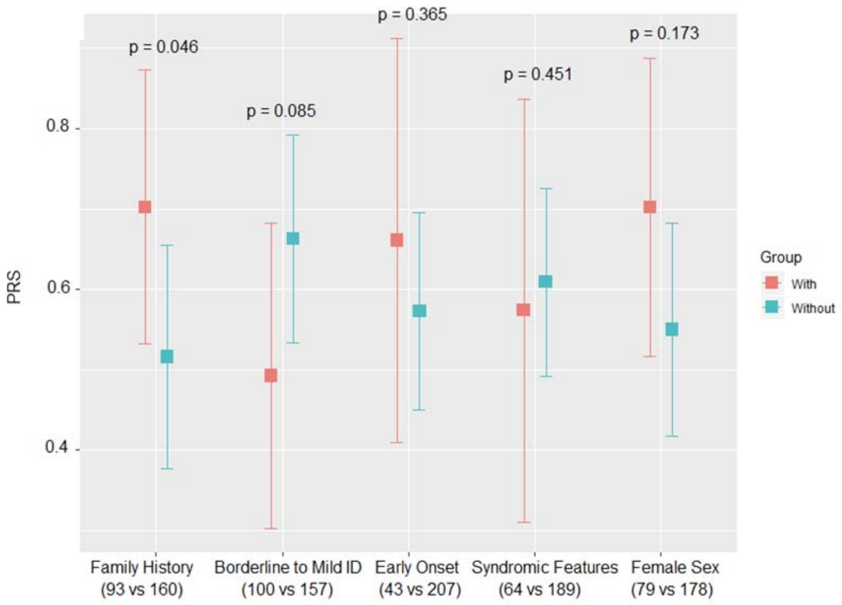

Figure S6. Box plots representing mean PRS for rare CNV subgroups of the schizophrenia cohort (n=257).

(**a**) PRS is not significantly different between individuals with or without the rare CNVs used in the study design (see Methods) or with or without the 28 clinically relevant CNVs and/or eleven high impact SNVs/indels ($p = 0.52$, Wilcoxon test, one-sided). (**b**) Examining five clinical/demographic variables (family history of schizophrenia/psychosis, ID, early age at onset, mild syndromic features, and biological sex); the mean common variant schizophrenia-related PRS at PT 0.05[37] was significantly higher in individuals with a positive family history of psychotic illness.

Number of individuals in each of the subgroups with and without the specified variants/features is indicated below the respective graph. Coloured box, with vertical bars representing 95% confidence intervals.

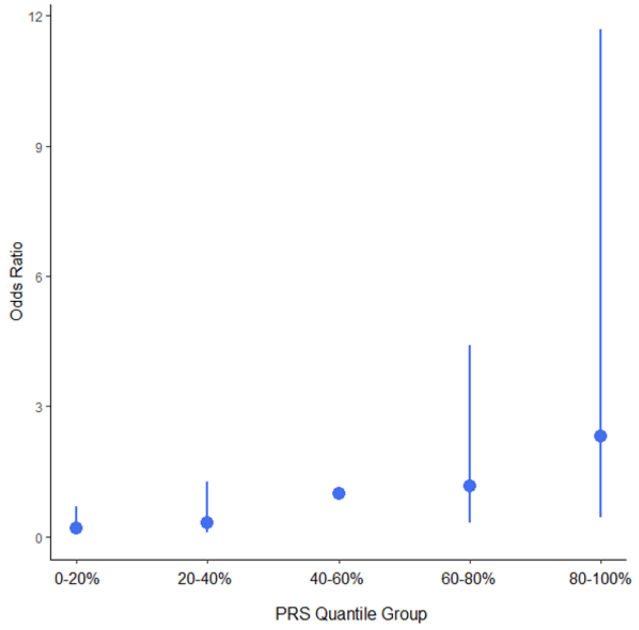

Figure S7. Odds ratios (OR) of schizophrenia at different PRS quantiles. Schizophrenia OR was calculated for schizophrenia and TOF individuals in five PRS percentiles (see Methods for details). Using pT=0.05, individuals within the top twentieth percentile of PRS had a 2.92-fold (95% CI: 1.05-8.11) increased OR for schizophrenia compared to those in other percentiles. The burden of rare clinically relevant variants (CNVs/SNVs/indels) was not significantly different between the 16 individuals in the top twentieth percentile group and others in the sample (Fisher's exact test OR: 0.97, CI: 0-3.78, p value: 0.66).

References:

1 C Yuen, R. K. *et al.* Whole genome sequencing resource identifies 18 new candidate genes for autism spectrum disorder. *Nat Neurosci* **20**, 602-611, doi:10.1038/nn.4524 (2017).

2 Reuter, M. S. *et al.* Haploinsufficiency of vascular endothelial growth factor related signaling genes is associated with tetralogy of Fallot. *Genet Med* **21**, 1001-1007, doi:10.1038/s41436-018-0260-9 (2019).

3 Layer, R. M., Chiang, C., Quinlan, A. R. & Hall, I. M. LUMPY: a probabilistic framework for structural variant discovery. *Genome Biology* **15**, R84, doi:10.1186/gb-2014-15-6-r84 (2014).

4 Chen, X. *et al.* Manta: rapid detection of structural variants and indels for germline and cancer sequencing applications. *Bioinformatics (Oxford, England)* **32**, 1220-1222, doi:10.1093/bioinformatics/btv710 (2016).

5 Trost, B. *et al.* A Comprehensive Workflow for Read Depth-Based Identification of Copy-Number Variation from Whole-Genome Sequence Data. *American journal of human genetics* **102**, 142-155, doi:10.1016/j.ajhg.2017.12.007 (2018).

6 *The Genome Aggregation Database (gnomAD)*, https://gnomad.broadinstitute.org/

7 *The Exome Aggregation Consortium (ExAC)*, http://exac.broadinstitute.org/

8 *The International Genome Sample Resource (1000 Genomes Project)*, https://www.internationalgenome.org/

9 Collins, R. L. *et al.* An open resource of structural variation for medical and population genetics. *bioRxiv*, 578674, doi:10.1101/578674 (2019).

10 Richards, S. *et al.* Standards and guidelines for the interpretation of sequence variants: a joint consensus recommendation of the American College of Medical Genetics and Genomics and the Association for Molecular Pathology. *Genetics in Medicine* **17**, 405-423, doi:10.1038/gim.2015.30 (2015).

11 Rees, E. *et al.* Analysis of Intellectual Disability Copy Number Variants for Association With Schizophrenia. *JAMA psychiatry* **73**, 963-969, doi:10.1001/jamapsychiatry.2016.1831 (2016).

12 Guilmatre, A. *et al.* Recurrent rearrangements in synaptic and neurodevelopmental genes and shared biologic pathways in schizophrenia, autism, and mental retardation. *Archives of general psychiatry* **66**, 947-956, doi:10.1001/archgenpsychiatry.2009.80 (2009).

13 McCarthy, S. E. *et al.* De novo mutations in schizophrenia implicate chromatin remodeling and support a genetic overlap with autism and intellectual disability. *Molecular psychiatry* **19**, 652-658, doi:10.1038/mp.2014.29 (2014).

14 Sebat, J., Levy, D. L. & McCarthy, S. E. Rare structural variants in schizophrenia: one disorder, multiple mutations; one mutation, multiple disorders. *Trends Genet* **25**, 528-535, doi:10.1016/j.tig.2009.10.004 (2009).

15 Damaj, L. *et al.* CACNA1A haploinsufficiency causes cognitive impairment, autism and epileptic encephalopathy with mild cerebellar symptoms. *European journal of human genetics : EJHG* **23**, 1505-1512, doi:10.1038/ejhg.2015.21 (2015).

16 O'Brien, J. E. & Meisler, M. H. Sodium channel SCN8A (Nav1.6): properties and de novo mutations in epileptic encephalopathy and intellectual disability. *Frontiers in genetics* **4**, 213, doi:10.3389/fgene.2013.00213 (2013).

17 Carney, R. M. *et al.* Identification of MeCP2 mutations in a series of females with autistic disorder. *Pediatric neurology* **28**, 205-211, doi:10.1016/s0887-8994(02)00624-0 (2003).

18 Lehman, A. *et al.* Loss-of-Function and Gain-of-Function Mutations in KCNQ5 Cause Intellectual Disability or Epileptic Encephalopathy. *American journal of human genetics* **101**, 65-74, doi:10.1016/j.ajhg.2017.05.016 (2017).

19 Yan, K. *et al.* Mutations in the Chromatin Regulator Gene BRPF1 Cause Syndromic Intellectual Disability and Deficient Histone Acetylation. *American journal of human genetics* **100**, 91-104, doi:10.1016/j.ajhg.2016.11.011 (2017).
20 Fassio, A. *et al.* SYN1 loss-of-function mutations in autism and partial epilepsy cause impaired synaptic function. *Human Molecular Genetics* **20**, 2297-2307, doi:10.1093/hmg/ddr122 (2011).
21 Wallace, R. H. *et al.* Febrile seizures and generalized epilepsy associated with a mutation in the Na+-channel ß1 subunit gene SCN1B. *Nature Genetics* **19**, 366-370, doi:10.1038/1252 (1998).
22 Durand, C. M. *et al.* Mutations in the gene encoding the synaptic scaffolding protein SHANK3 are associated with autism spectrum disorders. *Nature Genetics* **39**, 25, doi:10.1038/ng1933 (2006).
23 Giliberti, A. *et al.* MEIS2 gene is responsible for intellectual disability, cardiac defects and a distinct facial phenotype. *European Journal of Medical Genetics*, doi:10.1016/j.ejmg.2019.01.017 (2019).
24 Ambalavanan, A. *et al.* De novo variants in sporadic cases of childhood onset schizophrenia. *European Journal Of Human Genetics* **24**, 944, doi:10.1038/ejhg.2015.218 (2015).
25 Fromer, M. *et al.* De novo mutations in schizophrenia implicate synaptic networks. *Nature* **506**, 179-184, doi:10.1038/nature12929 (2014).
26 Tsankova, N., Renthal, W., Kumar, A. & Nestler, E. J. Epigenetic regulation in psychiatric disorders. *Nature Reviews Neuroscience* **8**, 355-367, doi:10.1038/nrn2132 (2007).
27 Costain, G. *et al.* Pathogenic rare copy number variants in community-based schizophrenia suggest a potential role for clinical microarrays. *Human molecular genetics* **22**, 4485-4501, doi:10.1093/hmg/ddt297 (2013).
28 Lowther, C. *et al.* Impact of IQ on the diagnostic yield of chromosomal microarray in a community sample of adults with schizophrenia. *Genome medicine* **9**, 105, doi:10.1186/s13073-017-0488-z (2017).
29 Kearney, H. M., Thorland, E. C., Brown, K. K., Quintero-Rivera, F. & South, S. T. American College of Medical Genetics standards and guidelines for interpretation and reporting of postnatal constitutional copy number variants. *Genet Med* **13**, 680-685, doi:10.1097/GIM.0b013e3182217a3a (2011).
30 Bergen, S. E. *et al.* Genetic modifiers and subtypes in schizophrenia: investigations of age at onset, severity, sex and family history. *Schizophr Res* **154**, 48-53, doi:10.1016/j.schres.2014.01.030 (2014).
31 Bigdeli, T. B. *et al.* Genome-wide association study reveals greater polygenic loading for schizophrenia in cases with a family history of illness. *American journal of medical genetics. Part B, Neuropsychiatric genetics : the official publication of the International Society of Psychiatric Genetics* **171b**, 276-289, doi:10.1002/ajmg.b.32402 (2016).
32 Woolston, A. L. *et al.* Genetic loci associated with an earlier age at onset in multiplex schizophrenia. *Sci Rep* **7**, 6486-6486, doi:10.1038/s41598-017-06795-8 (2017).
33 Bouwkamp, C. G. *et al.* Copy Number Variation in Syndromic Forms of Psychiatric Illness: The Emerging Value of Clinical Genetic Testing in Psychiatry. *The American journal of psychiatry* **174**, 1036-1050, doi:10.1176/appi.ajp.2017.16080946 (2017).
34 McDonald-McGinn, D. M. *et al.* 22q11.2 deletion syndrome. *Nature Reviews Disease Primers* **1**, 15071, doi:10.1038/nrdp.2015.71 (2015).
35 Dolcetti, A. *et al.* 1q21.1 Microduplication expression in adults. *Genet Med* **15**, 282-289, doi:10.1038/gim.2012.129 (2013).
36 Priori, S. G. *et al.* Mutations in the cardiac ryanodine receptor gene (hRyR2) underlie catecholaminergic polymorphic ventricular tachycardia. *Circulation* **103**, 196-200, doi:10.1161/01.cir.103.2.196 (2001).

37 Schizophrenia Working Group of the Psychiatric Genomics Consortium *et al.* Biological insights from 108 schizophrenia-associated genetic loci. *Nature* **511**, 421, doi:10.1038/nature13595 (2014).

38 Euesden, J., Lewis, C. M. & O'Reilly, P. F. PRSice: Polygenic Risk Score software. *Bioinformatics (Oxford, England)* **31**, 1466-1468, doi:10.1093/bioinformatics/btu848 (2015).

39 Dolzhenko, E. *et al.* Detection of long repeat expansions from PCR-free whole-genome sequence data. *Genome Res* **27**, 1895-1903, doi:10.1101/gr.225672.117 (2017).

40 Tanaka, E., Maruyama, H., Morino, H. & Kawakami, H. Detection of large expansions in SCA8 using a fluorescent repeat-primed PCR assay. *Hiroshima journal of medical sciences* **60**, 63-66 (2011).

41 Singh, T. *et al.* The contribution of rare variants to risk of schizophrenia in individuals with and without intellectual disability. *Nature Genetics* **49**, 1167-1173, doi:10.1038/ng.3903 (2017).

42 Genovese, G. *et al.* Increased burden of ultra-rare protein-altering variants among 4,877 individuals with schizophrenia. *Nat Neurosci* **19**, 1433-1441, doi:10.1038/nn.4402 (2016).

43 Ware, J. S., Samocha, K. E., Homsy, J. & Daly, M. J. Interpreting de novo Variation in Human Disease Using denovolyzeR. *Current protocols in human genetics* **87**, 7.25.21-27.25.15, doi:10.1002/0471142905.hg0725s87 (2015).

44 Estruch, S. B. *et al.* Proteomic analysis of FOXP proteins reveals interactions between cortical transcription factors associated with neurodevelopmental disorders. *Hum Mol Genet* **27**, 1212-1227, doi:10.1093/hmg/ddy035 (2018).

45 Hakimi, M. A., Dong, Y., Lane, W. S., Speicher, D. W. & Shiekhattar, R. A candidate X-linked mental retardation gene is a component of a new family of histone deacetylase-containing complexes. *The Journal of biological chemistry* **278**, 7234-7239, doi:10.1074/jbc.M208992200 (2003).

46 Gocke, C. B. & Yu, H. ZNF198 stabilizes the LSD1-CoREST-HDAC1 complex on chromatin through its MYM-type zinc fingers. *PloS one* **3**, e3255, doi:10.1371/journal.pone.0003255 (2008).

47 Connaughton, D. M. *et al.* Mutations of the Transcriptional Corepressor ZMYM2 Cause Syndromic Urinary Tract Malformations. *American journal of human genetics* **107**, 727-742, doi:10.1016/j.ajhg.2020.08.013 (2020).
